# Leveraging long short-term memory (LSTM)-based neural networks for modeling structure–property relationships of metamaterials from electromagnetic responses

**DOI:** 10.1038/s41598-021-97999-6

**Published:** 2021-09-20

**Authors:** Prajith Pillai, Parama Pal, Rinu Chacko, Deepak Jain, Beena Rai

**Affiliations:** TCS Research, Tata Consultancy Services, Mumbai, India

**Keywords:** Metamaterials, Metamaterials

## Abstract

We report a neural network model for predicting the electromagnetic response of mesoscale metamaterials as well as generate design parameters for a desired spectral behavior. Our approach entails treating spectral data as time-varying sequences and the inverse problem as a single-input multiple output model, thereby compelling the network architecture to learn the geometry of the metamaterial designs from the spectral data in lieu of abstract features.

## Introduction

Metamaterials (MMs) are artificial, mesostructured multi-material systems which demonstrate highly engineerable electromagnetic (EM) responses when excited by a suitable stimulus. The capability to arbitrarily program spectral responses, more specifically linear and nonlinear EM properties in response to external fields, arises from patterning these artificial composites as periodic 2D or 3D arrays of subwavelength conducting elements or unit cells. These unit cells (also known as ‘meta atoms’), are spaced by distances that are of the order of a fraction of the interrogating wavelength within the spectral region of interest (anywhere from the microwave to visible). This enables the manipulation of target functionalities such as the effective electrical permittivity and effective magnetic permeability in a manner not supported in naturally occurring materials^1–3^. This unique customizability leads to phenomena such as negative refractive indices and resonant phenomena at desired wavelengths, thereby opening up new frontiers for powerful photonic tools for imaging^4^, sensing^5^, wavefront control^6^, and nonlinear optical generation^7^.

Complex fabrication methods for tailored control of electric and magnetic properties demand that metamaterial device topologies be preemptively articulated^6,8–10^. Traditional MM design processes rely heavily on the designer’s knowledge and intuition and typically commence with an initial candidate geometry. The spectral behavior of this preliminary design is calculated by solving Maxwell's equations via standard numerical techniques such as the finite-difference time domain (FDTD) method, finite element method (FEM), and the boundary element method (BEM). A mismatch between the desired response and the preliminary design performance entails tweaking geometrical and material parameters iteratively till a satisfactory outcome is achieved. These iterations typically involve large, time-consuming trial-and-error searches over complex candidate geometries and configurations. In recent times, commercially available electromagnetic solver software packages have been widely adopted by the MM community for designing metamaterials but there too, only a fraction of candidate geometries can be explored and is largely dependent on the designer’s intuitive reasoning. Of late, numerous studies have been directed towards the selection of a metamaterial design algorithm that searches vast parameter spaces for a best-fit solution to achieve performance characteristics in a manner that is efficient and computationally inexpensive^11,12^. This has led to the development, optimization and implementation of data-driven MM design frameworks as an area of active research. Deep learning-based models which compute abstract representations of data by learning via multiple layers of nonlinear transformations, have been the go-to approach. These models typically require curated datasets consisting of metamaterial geometries and their EM responses for training^11,13–15^. An efficient MM design scheme encompasses two aspects: the forward problem (predicting the EM response for a given geometry) as well as the inverse problem (generating the structural parameters for a desired EM response). The forward model is viewed akin to a prototyping method that avoids rule-based methods of numerically solving Maxwell’s equations whereas an inverse model presents a viable paradigm to arrive at a design that captures user-specified spectral properties.

In this work, we explore the unique property of recurrent neural networks (RNNs)^16–18^ to leverage the inherent sequential nature of the spectral responses. We base our methodology on deep learning models that have been used for solving one-to-many sequence problems e.g. image captioning, wherein the image information is provided as an input to either a convolutional neural network (CNN) or a multilayer perceptron (MLP). The NN (CNN or MLP) then passes the image information to an RNN (typically, long short term memory (LSTM)) in the form of features and corresponding captions are generated based on the information from the current timestep as well as the previous timesteps. We leverage the ability of LSTMs for learning long-term dependencies for retaining the sequential features through memory cells. The spectral sequence passed through the input gate of the LSTM extracts the most important features and discards the unimportant features through forget gates. LSTM networks have been widely used in sequence-to-sequence learning tasks which is essentially how we pose our spectrum-design-spectrum problem^19^.

A convolutional neural network (CNN) may also perform well for the problem statement and dataset at hand, although a detailed comparison is out of the scope of this current work. Spectral responses may be discretized as per the user’s requirement (depending on how the Maxwell’s equations are solved using numerical methods) thereby resulting in sequences of variable lengths and the model will still be able to perform inverse predictions, owing to the unique advantage(s) offered by RNNs.

**Figure 1:**
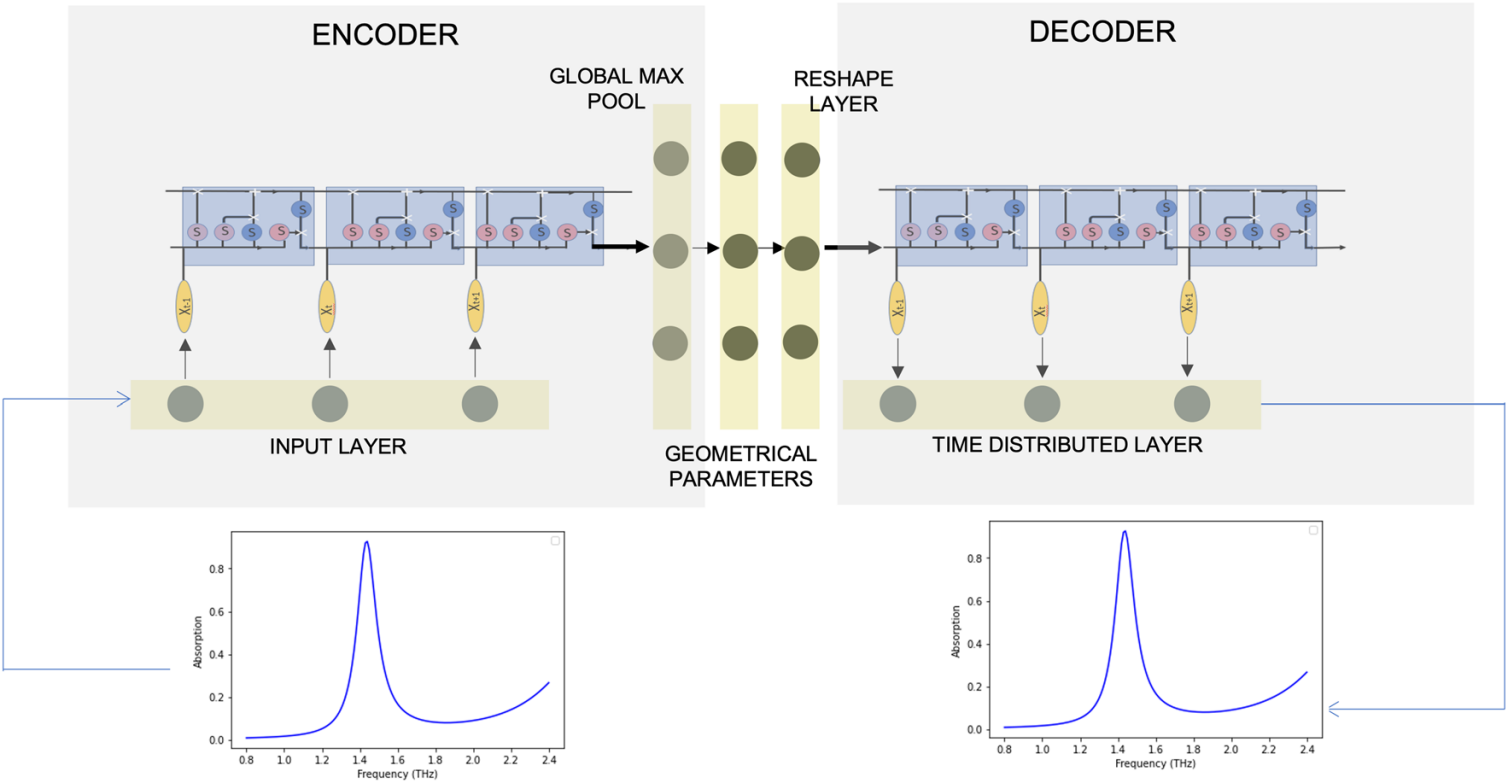
Structure of the deep learning model for deriving structure-property relationships of metamaterials. The architecture represents a tandem network comprising an inverse design network connected to a forward modeling network in an encoder-decoder configuration for predicting the geometrical dimensions by providing the absorption spectra as the input. Geometrical design parameters are obtained from the intermediate layer of the network structure.

We report a dual-model framework comprising a forward model for predicting the response spectra for a given set of design parameters (here, geometrical dimensions) and a tandem network for generating design parameters corresponding to a desired EM response. Our forward model is trained using geometry as the input and the corresponding EM spectral response as the output, and we subsequently pose the training for the inverse problem as a single-input, multi-output (SIMO) model using a tandem network of an encoder and a decoder with sequential input data (EM responses). Our training approach for the tandem network is similar to autoencoder architectures used for dimensionality reduction and learning latent representations^20^. The dimensions are obtained as intermediate outputs of the trained inverse model. Automating the solution of inverse problems, i.e., starting with a desired spectral behavior and working backwards to arrive at its corresponding MM topology, is often a challenge due to the observation of *near-identical* spectra for distinct device geometries. This may lead to a one-to-many mapping scenario between the EM response space and the design space, i.e., more than one design may yield almost completely overlapping response spectra^21,22^. We address this complexity by using a tandem encoder-decoder architecture as shown in Fig. 1 which leverages the powerful internal architecture of LSTMs to identify relevant information from long, sequential data. We apply our architecture to a broadband, near ‘perfect’ metamaterial absorber designed to operate in the terahertz spectral regime.

## Results

**Figure 2:**
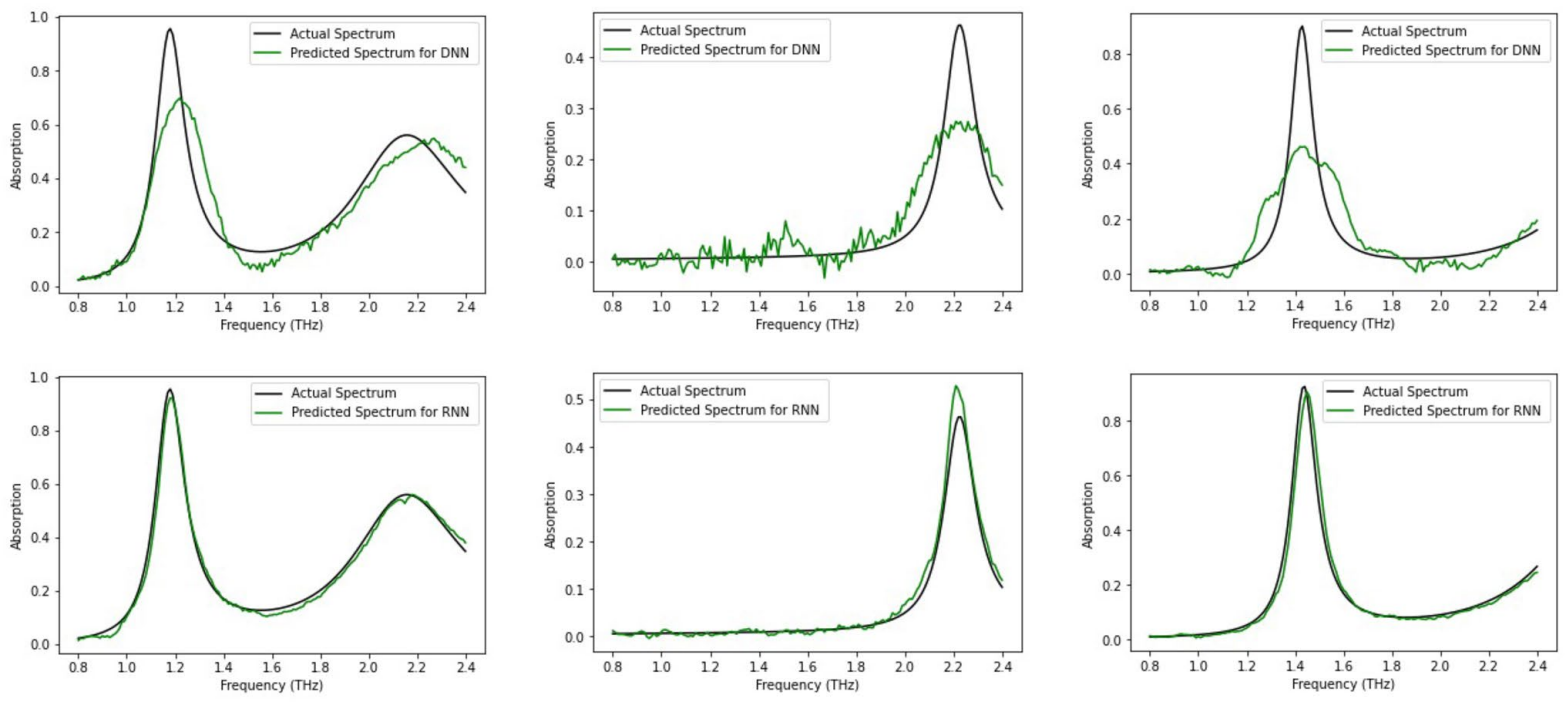
Network predictions of the frequency dependent absorption spectra (green curves) and the simulated spectra (black curves). The plots in the top row show examples of structure–property prediction results in comparison with a DNN-only forward model whereas the bottom row depict the forward prediction results of the same set of examples using the proposed RNN (LSTM) model.

The goal is to develop a design framework that can extract precise MM structural dimensions for a given target response. More specifically, we applied our framework to a metamaterial film composed of individual split-ring resonators (SRRs) which has been designed for near-unity absorption over a range of terahertz wavelengths (see “Methods”). The training for the forward model took approximately 6 h on a standard Intel Core i5 CPU @1.5 GHz with 8 GB of RAM. In order to validate our hypothesis, namely, efficient learning using LSTMs by treating the spectral response as a sequence of absorption values, we compare our model’s predictions to a baseline architecture using a feed forward neural network without an LSTM. Figure 2 clearly depicts the superiority of the LSTM-based forward network in predicting the spectral response. We hypothesize that architectures consisting solely of networks of fully connected multi-layer perceptrons with the geometrical parameters as generic features, may be learning some dependency (order) between the layers. However, they are unable to efficiently leverage the inherent sequential nature of the data. This contrasts with the manner in which LSTMs tackle sequential data owing to their superior architecture. Therefore, for the inverse model as well, the LSTM layer is a fitting choice considering the sequential nature of the input spectral data. We checked the precision of our forward model’s predictions via comparison with ground truth data generated by ‘virtual’ experiments wherein we solved for Maxwell’s equations (for the geometry under consideration) and computed the corresponding spectral performance using FEM simulations. The overall objective function for the tandem network was to minimize the sum of the losses from the encoder output (geometry) and the decoder output (spectra). In other words, while training, the tandem network learns to yield the dimensional information for the SRR structures while attempting to reproduce the spectral input given. This necessitates the network to learn a latent representation of input spectra that closely resembles the desired geometry. Table 1 summarizes the mean square error (MSE) losses for the forward and tandem model. One may argue that an encoder-only model will be sufficient for mapping the spectra to the dimensions however a comparison between an encoder-only and a tandem shows that the tandem performs better when trained with a combined loss albeit at the cost of slightly higher computational times (Table S3). The tandem approach also allows for modification of the encoder architecture to incorporate multiple data forms (electric field values, topological images, field profiles etc.).

**Table 1:** Mean squared error (MSE) losses for the tandem and forward model.

	Train	Validation	Test
Geometrical dimensions (tandem model)	144.58	135.29	143.92
Forward Model	0.00366	0.00375	0.00372

In order to further illustrate the performance of our model, we randomly selected two cases from our test set and compared the predicted geometrical values vs the ground truth (Table 2).

**Table 2:** Predicted vs ground truth (actual) values of SRR geometrical parameters.

	D1				D2			
	*l*	*w*	*t*	*c*	*l*	*w*	*t*	*c*
Predicted values	22.49	3.99	9.62	10.51	27.46	5.39	7.88	10.73
Ground truth	23.90	4.75	9.00	11.50	27.90	5.25	8.10	10.60

All values are in micrometers. D1 and D2 are randomly selected geometries from the test set.

The results validate our architecture as an efficient and accurate model for the design and discovery of complex metamaterial structures. As a qualitative indicator of computational efficiency, the COMSOL full-wave simulations for ~ 500 designs took around 54 h on a standard Intel Core i5-8250U CPU @ 1.60 GHz with 8 GB of RAM; our model took approximately 6 h to train (for a 1000 epochs) and less than 0.5 s to predict spectra for the 463 distinct designs in the test set. MM devices, such as the broadband absorber considered by us, have vast scope for a multitude of applications across domains as diverse as high-speed communications, photovoltaics, novel healthcare devices, stealth applications for defense amongst others, however, their technology penetration has been limited so far due to complex design processes that require advanced skills and knowledge base(s). The advent of data-driven models makes it possible to generate designs based on user-defined, on-demand device performances within fractional time durations.

## Methods

### Preparing the training data

**Figure 3:**
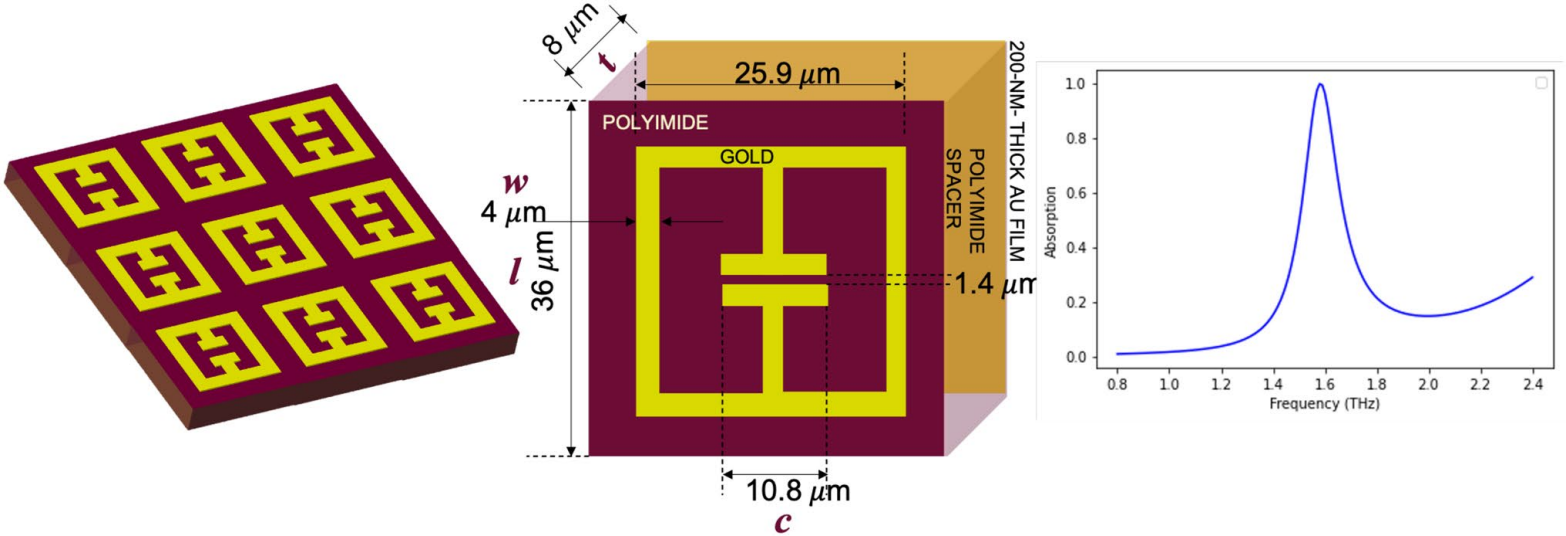
Schematic of a terahertz dielectric absorber comprising of a dual metallic and a dual dielectric layer structure^23^ composed of split-ring resonator unit cells arranged in a regularly spaced array (left). Dimensional parameters of an SRR unit cell (center) of a metamaterial optimized to demonstrate near-unity absorption at 1.6 THz (right).

Our candidate design is a metamaterial film optimized for near-unity absorption at 1.6 THz for a wide range of incidence angles^23^. This is achieved by arranging gold split-ring resonators (SRRs) in a periodic array on a dielectric substrate. Split-ring resonators (SRRs) are a widely studied metamaterial element which have negative magnetic permeabilities thereby accommodating a wide range of practical devices such as filters, modulators, switches, amongst others. As the name suggests, SRRs support inductive-capacitive (LC) resonances, which arise primarily from a capacitive gap (or ‘split’) in the resonator’s perimeter combined with the symmetry of the circulating current loops along the outer perimeter. The absorber design comprises two metallic layers on either side of an 8-micron thick dielectric polyimide spacer wherein the top layer contains the lattice of square-shaped, 200-nm thick gold SRRs and the bottom layer is a 200-nm thick continuous film of gold. Figure 3 depicts a schematic of the device along with its dimensions. The dimensions of the SRRs, defined chiefly via four parameters, namely, the linewidth of the gold features (*w*), the thickness of the dielectric substrate (*t*), the length of the gold layer (*l*) and the width of the capacitor region (*c*), dictate the spectral response of the absorber. We use a commercially available numerical package (COMSOL Multiphysics 5.3) to compute full-wave EM simulations for obtaining the spectral response of our device by solving Maxwell’s equations using the finite element method with appropriate boundary conditions. On excitation with a linearly polarized wave, we observe a large absorption peak centered at 1.6 THz as shown in Fig. 3.

We subsequently populated our dataset by generating variants of this design through parametric sweeps of the unit cell dimensions (*w*, *t*, *l*, *c*) and computing the corresponding EM response curves, each of which were discretized into 161 data points. A total of 5163 geometries were modeled out of which 3091 conformed to SRR morphologies and demonstrated spectral responses with characteristic resonance peak(s) whereas the remaining variants were treated as geometric outliers and subsequently omitted. Our resultant geometry-spectrum dataset was subsequently split into training, validation, and test sets in the ratio 0.7:0.15:0.15.

### Neural network structure

The forward model is a combination of a multilayer perceptron (MLP) and a bidirectional long short-term memory (LSTM) layer wherein the inputs comprise of 16 features obtained by combining the four geometrical parameters (‘*l*’, ‘*t’*, ‘*w’*, ’*c’*) using ratios and products. The DNN has two hidden layers with 161 neurons and 500 neurons respectively. Additional network parameters are mentioned in Table S1 of the supplementary document. The LSTM forward model has a single dense layer with 161 neurons and a bidirectional LSTM layer with 200 units. We studied the change in the loss of the DNN-only baseline model as a function of the number of input features (Table S2) and we observed that the lowest losses were obtained for the case of the 16 expanded dimensions as the input. In order to be consistent and perform a comparison with the baseline model, we retained these dimensions as the input features for our final network model as well. A dense layer is connected to a bidirectional LSTM using a reshape layer and the final output is then obtained through a time-distributed wrapper on the final feed-forward layer with a linear activation function. The reshape layer and the time-distributed wrapper serve to align the dimensions of the LSTM and MLP layers. The regularization parameter and the learning rate (along with the Adam optimizer) was set at 0.00001 and 0.0001 respectively. The architecture was trained for 1000 epochs and the mean squared error (MSE) was selected as the loss function.

Once successfully trained, the forward model was connected to an inverse model to form a tandem neural network which used a single input multiple output (SIMO) model. The tandem network consisted of an encoder and a decoder, wherein the decoder had the weights saved from the pre-trained forward model. We treat the 161-dimensional absorption spectra as the sequential input and output to the encoder-decoder architecture. The geometrical features are the desired outputs which are obtained from the last layer of the proposed encoder. The encoder input sequences encompassing the architecture for the inverse predictions are encoded to the MM geometry using a combination of the LSTM layer and an MLP. Within the encoder, the MLP transforms the LSTM output averaged over the timesteps into the dimensions (geometry). The network is forced to learn the geometry by applying a loss function to the encoder architecture. The loss function is selected as MSE for both the outputs and trained for 1000 epochs. The mean absolute error on the predicted geometrical parameters in the test data set is 1.0195, 0.60492, 0.66194, 0.5482 for *l*, *w*, *t*, and *c* respectively. We performed the fivefold cross validation to ensure that the models are generalized.

## Supplementary Information

Supplementary Information.

## Data Availability

All data generated during this study are available from the authors on reasonable request.
