# Supplementary material for: Leveraging long short-term memory (LSTM)-based neural networks for modeling structure–property relationships of metamaterials from electromagnetic responses
Source: Sci Rep. 2021 Sep 20;11:18629. doi: 10.1038/s41598-021-97999-6 (PMC8452651; doi:10.1038/s41598-021-97999-6)
Supplement: Supplementary file 1 — Supplementary Information. [file 41598_2021_97999_MOESM1_ESM.docx]

Leveraging Long Short-Term Memory (LSTM)-based neural networks for modeling structure-property relationships of metamaterials from electromagnetic responses

Prajith Pillai, Parama Pal, Rinu Chacko, Deepak Jain, and Beena Rai

TCS Research, Tata Consultancy Services, India

**Table S1:** Hyperparameter Range for DNN forward model

| **S. No.** | **Hyperparameter** | **Range** |
| --- | --- | --- |
| 1 | Learning Rate | [10^-1^ -10^-5^ ] |
| 2 | Regularization Type | [L1,L2] |
| 3 | Regularization parameter | [10^-1^ -10^-5^ ] |
| 4 | Neurons | [100-1000] |
| 5 | Activation Function | [Tanh, Sigmoid, Relu] |

**Table S2:** Number of input features vs losses for the DNN model

| **Input Features** | **Features** | **DNN train loss** | **DNN val loss** | **DNN test loss** |
| --- | --- | --- | --- | --- |
| 4 | length(l), width(w), substrate thickness(t) and capacitor width(c). | 0.01562 | 0.016105 | 0.01544 |
| 8 | l, w, t, c, l*w, l*t, l*c, w*t | 0.005102 | 0.005311 | 0.005204 |
| 16 | l, w, t, c, l*w, l*t, l*c, w*t, w*c, t*c, l/w, l/t, l/c, w/t, w/c, t/c | 0.004094 | 0.004438 | 0.004291 |
| 32 | l, w, t, c, l*w, l*t, l*c, w*t, w*c, t*c, l/w, l/t, l/c, w/t, w/c, t/c, e^(l, w, t, c), log(l,w,t,c),((l*w)+(t,c)),((t*c)+(l,w)),((t*w)+(l,c)),((l*c)+(t,w) ) ) | 0.041648 | 0.041839 | 0.04046 |

**Table S3:** Loss and computational time comparison for encoder-only, tandem with non-trainable weights and biases, and tandem with trainable weights and biases

| **Architecture** | **Loss** | **Loss (Dimensions)** | **Loss (Spectrum)** | **Val Loss** | **Val Loss (dimensions)** | **Val Loss (spectrum)** | **Time per iteration ( 16GB RAM, Intel Core i7-9750H CPU @ 2.60GHz)** |
| --- | --- | --- | --- | --- | --- | --- | --- |
| Tandem (trainable weights) | 144.58 | 144.54 | 0.0071 | 135.29 | 135.25 | 0.0059 | 24s |
| Encoder-only | 174.66 |  |  | 161.43 |  |  | 9s |
| Tandem (non-trainable weights) | 197.85 | 197.78 | 0.029 | 179.03 | 178.96 | 0.0298 | 24s |

**Tandem with non-trainable weights and biases:** The decoder weights were fixed with the pre-trained forward model

**Tandem with trainable weights and biases:** The decoder weights were initialized with the pre-trained forward model but were allowed to change during the training
